# Shedding light on the bacterial resistance to toxic UV filters: a comparative genomic study

**DOI:** 10.7717/peerj.12278

**Published:** 2021-11-01

**Authors:** Clément Lozano, Philippe Lebaron, Sabine Matallana-Surget

**Affiliations:** 1 Division of Biological and Environmental Sciences, Faculty of Natural Sciences, University of Stirling, Stirling, United Kingdom; 2 Sorbonne Université, CNRS, Laboratoire de Biodiversité et Biotechnologies Microbiennes, USR3579, Observatoire Océanologique, Sorbonne Université, Banyuls-sur-mer, France

**Keywords:** UV filters, Marine bacteria, Genomic comparison

## Abstract

UV filters are toxic to marine bacteria that dominate the marine biomass. Ecotoxicology often studies the organism response but rarely integrates the toxicity mechanisms at the molecular level. In this study, *in silico* comparative genomics between UV filters sensitive and resistant bacteria were conducted in order to unravel the genes responsible for a resistance phenotype. The genomes of two environmentally relevant Bacteroidetes and three Firmicutes species were compared through pairwise comparison. Larger genomes were carried by bacteria exhibiting a resistant phenotype, favoring their ability to adapt to environmental stresses. While the antitoxin and CRISPR systems were the only distinctive features in resistant Bacteroidetes, Firmicutes displayed multiple unique genes that could support the difference between sensitive and resistant phenotypes. Several genes involved in ROS response, vitamin biosynthesis, xenobiotic degradation, multidrug resistance, and lipophilic compound permeability were shown to be exclusive to resistant species. Our investigation contributes to a better understanding of UV filters resistance phenotypes, by identifying pivotal genes involved in key pathways.

## Introduction

UV filters are active ingredients of sunscreens products, accounting for up to 30% in sun lotion formulation. Increasing tourism results in their direct and indirect discharge in aquatic biota, through bathing and wastewater treatment plants (Tovar-Sánchez, Sánchez-Quiles & Rodríguez-Romero, 2019). Their bioaccumulation, biomagnification (Lozano et al., 2020a) and their occurrence were reported in freshwater and marine environments from the surface microlayer (Fagervold et al., 2019), water column (Langford et al., 2015; Sánchez Rodríguez, Rodrigo Sanz & Betancort Rodríguez, 2015) to sediments (Apel, Joerss & Ebinghaus, 2018; Ramos et al., 2015). UV filters toxicity was demonstrated on a broad range of organisms (Barone et al., 2019; Danovaro et al., 2008; Downs et al., 2016; He et al., 2019; Seoane et al., 2017; Stien et al., 2021; Ziarrusta et al., 2018), including bacteria (Lozano et al., 2020b), yet UV filters toxicity mechanisms remain unclear. Bacteria are an interesting ecotoxicological model as they are easy to handle, constitute a major part of the marine biomass (Bar-On, Phillips & Milo, 2018), and support essential function in marine ecosystems (Yilmaz et al., 2016). In a former study, marine bacteria from the major phyla, namely Actinobacteria, Bacteroidetes, Firmicutes, and Proteobacteria, were sensitive to UV filters from 200 µg/L (Lozano et al., 2020a). Environmentally relevant bacteria investigated in this study belong to the Bacteroidetes, the most abundant phyla after Proteobacteria in marine environments (Coclet et al., 2019), and the Firmicutes, widespread symbionts in marine organisms (Li et al., 2018; Math et al., 2010).

A growing number of bioinformatic tools designed to analyze and compare microbial genomes are available (Karp et al., 2019). They implement genome annotation tools, *e.g*. RAST, allowing for genome categorization and gene function identification. Functional genomic comparison is widely used to investigate variations between genomes or to depict particular traits (Kube et al., 2013; Wagner et al., 2019). For instance, Kube et al. (2013) provided an extensive functional analysis of the bacterium *Oleispira antarctica*, corroborating its capacity for alkane degradation and cold adaptation (Kube et al., 2013). Our study aims to perform a genomic comparison of UV filters sensitive and resistant species, using the RAST platform, and the subsystems hierarchical annotation scheme implemented in SEED. Bactericidal compounds can alter numerous cellular functions such as DNA replication, by targeting DNA gyrase (Lewin, Howard & Smith, 1991), membrane synthesis, by competing with lipid constituting the cell membrane (Müller et al., 2016), or protein synthesis, through binding to ribosomal subunits (Greulich et al., 2015). Although multiple genes might be responsible for bacterial resistance, the targets of UV filters or the membrane permeability towards these compounds are undetermined. Untargeted pairwise comparisons were conducted between resistant and sensitive species, in order to identify genes that could be involved in UV filters resistance and bridge the gap between genotypes and phenotypic traits. Comparisons were performed to address relevant functions, such as membrane biosynthesis, cell signaling, stress response and xenobiotic degradation.

## Materials and Methods

### Bacterial strain genomes

Genomes of bacterial species screened for sensitivity (Lozano et al., 2020a) were downloaded from NCBI (https://www.ncbi.nlm.nih.gov/genome). The following strains from comparable phyla and genera were selected based on their resistance profiles: *Paenibacillus glucanolyticus* NBRC 15330, *Bacillus megaterium* ATCC 14581, *Halobacillus dabanensis* CGMCC 1.3704, *Algoriphagus ornithinivorans* DSM 15282, and *Algoriphagus mannitolivorans* DSM 15301.

### Genome comparison

Bacterial genomes were uploaded on the RAST platform (https://rast.nmpdr.org). Subsequently, pairwise comparisons were conducted between sensitive and resistant bacteria belonging to the same Phylum (Table 1), using the SEED functional classification. Average nucleotide identity (ANI) between the compared species were calculated using the EZbiocloud platform (https://www.ezbiocloud.net) to provide a view of the species relativeness.

**Table 1:** Pairwise compared species and respective UV filters sensitivity.

Phyla	Species	Sensitive/resistant (UV filters)		ANI Score (%)
*Bacteroidetes*	*Algoriphagus mannitolivorans*	R	(HS)	72.13
*Bacteroidetes*	*Algoriphagus ornithinivorans*	S		
*Firmicutes*	*Paenibacillus glucanolyticus*	S	(EHMC)	65.32
*Firmicutes*	*Halobacillus dabanensis*	S	(EHMC, HS)	
*Firmicutes*	*Bacillus megaterium*	R	(EHMC, HS)	68.02
*Firmicutes*	*Halobacillus dabanensis*	S		

**Note:**

HS, Homosalate; EHMC, 2-Ethylexyl 4-methoxycinnamate.

### Protein comparison

Protein FASTA files were downloaded from the RAST platform and uploaded on the Orthovenn online tool (https://orthovenn2.bioinfotoolkits.net/) to generate Venn diagrams.

## Results and discussion

### Bacterial genome size as an indicator of bacterial resistance

Bacteroidetes selected in this study-belonging to the *Algoriphagus* genera-exhibited a smaller genome compared to Firmicutes (Table 2). Similarly, the number of rRNA, tRNA, and proteins was lower in the Bacteroidetes species. Among Firmicutes, *H*. *dabanensis* displayed the smaller genome with approximately 1,000 fewer proteins than *B. megaterium* and *P*. *glucanolyticus*. Noteworthy, UV filters resistant species for both Firmicutes and Bacteroidetes held the greater genome. A correlation between the appearance of resistance mechanisms and genome size has already been discussed earlier (Projan & Levy, 2007). Similarly, a high tRNA number has been correlated with temperature resistance (Siddhartha Sankar, Malay & Suvendra Kumar, 2010).

**Table 2:** Bacterial genomes structures.

Species	Chromosome	Plasmid	Size (Mb)	GC%	Genes	rRNA	tRNA	Proteins
Algoriphagus mannitolivorans*	1	0	4.15	42.7	3636	7	39	3564
*Algoriphagus ornithinivorans*	1	0	4.09	39.5	3,557	5	35	3,500
Paenibacillus glucanolyticus*	1	0	5.9	49.2	5,380	24	74	5,174
*Halobacillus dabanensis*	1	0	4.14	41.7	4,135	18	67	3,971
Bacillus megaterium*	1	6	5.34	38.1	5,541	41	123	5,268

**Note:**

Asterisks (*) indicate UV filters resistant bacteria.

**Figure 1:**
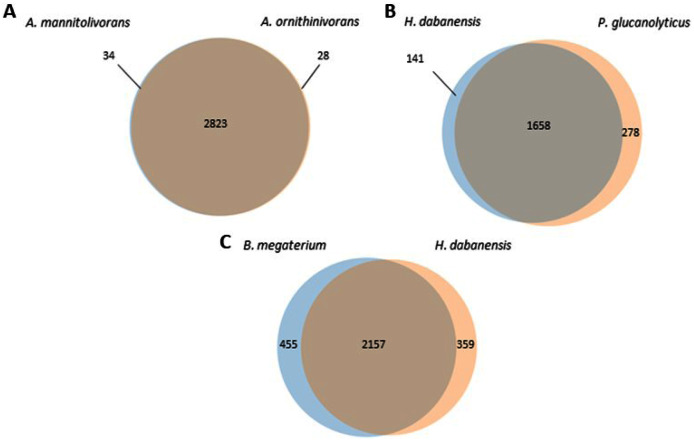
Venn diagrams representing the number of unique and shared proteins between (A) *A. mannitolivorans* and *A. ornithinivorans*, (B) *H. dabanensis* and *P. glucanolyticus*, (C) *B. megaterium* and *H. dabanensis*.

*A. mannitolivorans* and *A. ornithinivorans* shared 2,823 proteins and displayed 34 and 28 unique proteins, respectively (Fig. 1A). Among these proteins, *A. mannitolivorans* displayed two protein counts included in the “cellular aromatic compound metabolic process” cluster, identified as the 4, 5-DOPA dioxygenase extradiol, an enzyme involved in betalain biosynthesis. *P. glucanolyticus* and *H. dabanensis* shared 1,658 proteins (Fig. 1B). *B. megaterium* and *H. dabanensis* shared 2,157 proteins and exhibited 455 and 359 unique proteins, respectively (Fig. 1C). Interestingly bacteria displaying the greater number of unique proteins held the resistant phenotype.

### Comparative genomic analysis of *H. dabanensis* and *B. megaterium* revealed relevant resistance features

**Figure 2:**
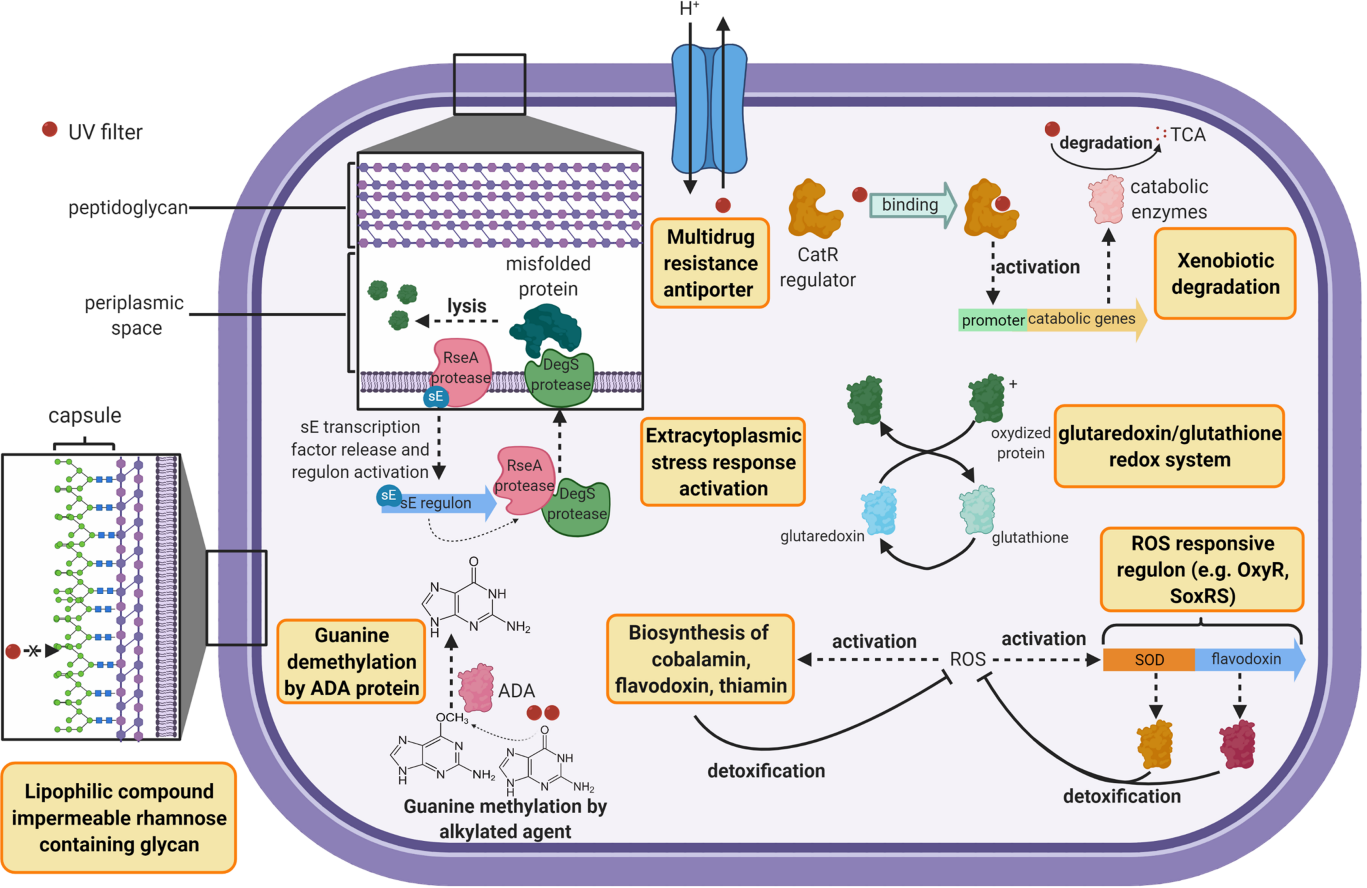
Cell diagram showing the hypothetical protective structures against UV filters, obtained from genomic functional comparison.

The cell wall constitutes the first barrier against xenobiotics. Therefore, genes classified in this functional category were compared in this study. *B. megaterium* displayed 14 unique genes belonging to the cell wall category while *H. dabanensis* had only one. Among them, the subsystems “Rhamnose containing glycans” and “sialic acid metabolism” were the most represented. Rhamnose is a six-carbon deoxy hexose, mostly incorporated in cell wall anchored polysaccharides, glycoproteins, and the capsule of many bacteria (Mistou, Sutcliffe & Van Sorge, 2016). It is essential for cell viability, environmental adaptation, and biofilm formation (Mäki & Renkonen, 2004; Michael et al., 2016). The presence of genes involved in rhamnose metabolism and the gene coding for the capsular polysaccharide biosynthesis protein strongly suggests that *B. megaterium* holds a capsule on its surface (see Fig. 2 for a schematic representation). While phospholipid bilayers are hypothetically permeable to lipophilic compounds such as homosalate, the capsule, made of a polysaccharides layer could prevent these compounds from entering the cell. Sialic acids are nine carbon sugar acids derivatives mainly incorporated into glycan chains. Common on the surface of pathogenic bacteria, they provide camouflage from the immune system by imitating eukaryotic structures.

Both species harbored genes involved in bacitracin response. *B. megaterium* possessed multiple stress response genes such as cadmium-transporting ATPase, multicopper oxidase and multidrug resistance transporter (Bcr/CflA family), the cytoplasmic copper homeostasis protein CutC, the mercuric resistance operon regulatory protein, and the membrane fusion protein of RND family multidrug efflux pump, known to confer resistance against multiple hydrophobic substrates (Nikaido & Takatsuka, 2009). Several gene copies belonging to the choline and betaine uptake and biosynthesis subsystem were observed in the genome of *B. megaterium*, including four choline specific ABC transporter. Choline is a precursor of glycine betaine, known as a potent osmoprotectant (Kiene, 1998). Furthermore, *B. megaterium* presented genes coding for flavodoxin, a small soluble protein belonging to the non-enzymatic antioxidant molecules, known to confer resistance against herbicides to soil bacteria (Coba de la Pena et al., 2013) (Fig. 2). Among the genes categorized in the regulation and cell signaling category, the aromatic hydrocarbon utilization transcriptional regulator CatR (LysR family) was the single unique gene belonging to *B. megaterium*. CatR regulates the expression of catechol and phenol degradation pathway, that has an aromatic structure (benzene) common to UV filters (Díaz & Prieto, 2000). Once the substrate binds to CatR, the promoter regulating the transcription of the catabolic genes is activated and the substrate is degraded into tricarboxylic acid intermediates (Díaz & Prieto, 2000) (Fig. 2).

### Comparative genome analysis of *H. dabanensis* and *Paenibacillus glucanolyticus*

Compared to the UV filter sensitive *H. dabanensis, P. glucanolyticus* harbored more genes coding for proteins involved in pyridoxine (vitamin B6), cobalamin (vitamin B12), and thiamin (vitamin B1) biosynthesis. Vitamins, such as vitamin B12 allow tolerance to oxidative stress in bacteria belonging to the Nitrospira phylum (Ferrer et al., 2016). Similarly to *B. megaterium*, *P. glucanolyticus* coded for Hyaluronan synthase and all genes belonging to the Rhamnose containing glycans subsystem, involved in capsule synthesis - a structure known to mediate antibiotic resistance (Campos et al., 2004). A total of 11 unique genes categorized in subsystem related to antibiotic and metal stress response were identified in the genomes of *P. glucanolyticus*. Among them, the Multidrug resistance transporter (Bcr/CflA family), reported being involved in bicyclomycin, fosfomycin, kanamycin, and sulfathiazole resistance (Smith, Kumar & Varela, 2009). Transcriptomic analyses highlighted that this transporter was overexpressed (4.35 fold change) in the presence of toluene (García et al., 2010) in *Pseudomonas putida*, hence revealing that this gene could play a pivotal role in xenobiotic resistance.

Both bacteria harbored DNA repair systems such as Rec, SOS response, and mutL-mutS system. *P. glucanolytics* expressed the ADA regulatory protein, involved in six-O-Methylguanine demethylation, a mechanism known to bypass G:C to A:T due to guanine methylation (Fig. 2). *P. glucanolyticus* genome contained multiple genes involved in response against oxidative stress such as superoxide dismutase, HtrA protease/chaperone protein, Glutaredoxin, and the outer membrane stress sensor protease DegS. HtrA protease/chaperone protein was shown to be involved in high temperature and oxidative stress tolerance in *Campylobacter jejuni* (Bæk et al., 2011). Mutation induced lack of glutathione-glutaredoxin in a *Rhodobacter* strain resulted in decreased growth rates and high sensitivity to oxidative stress (Li et al., 2004). In addition, proteomic analyses revealed that glutaredoxin was up-regulated in *Rhodobacter sp*. exposed to artificial UVB treatment (Pérez et al., 2017). Overall, we can postulate that among Firmicutes, both *P. glucanolyticus*, and *B. megaterium* were better fitted to cope with the potentially harmful effect of xenobiotics, including UV filters, than *H. dabanensis*.

### *Algoriphagus* displayed fewer genomics differences but different phenotypes

The UV filter resistant *A. mannitolivorans* showed a chromosomic Doc/Phd toxin-antitoxin system, known to help cells maintaining genome integrity over generations, control cell growth, and face environmental stresses, by entering into persistent states (Liu et al., 2008). Interestingly, *A. mannitolivorans* genome harbored the gene coding for the antitoxin ParD without the corresponding DNA gyrase targeting toxin, ParE. Therefore, we could hypothesize that ParD can compete WITH for other DNA gyrase binding molecules. DNA repair systems were similar between the two species. However, *A. ornithinivorans* showed five genes belonging to the CRISPR system, involved in bacterial prophage immunity.

*A. ornithinivorans* and *A. mannitolivorans* showed genes involved in the resistance against Beta-lactam, Bacitracin, Fosfomycin, fluoroquinolones, and metal such as cadmium, cobalt, and copper. Genes coding for multiple multidrug resistance efflux pumps were present in both species. Minor differences were observed between genes classified into the cell wall and capsule category. No genes involved in xenobiotics resistance were identified in the two strains. As discussed earlier, these two species displayed only a few unique proteins (Fig. 1A). Overall, these observations suggested that only minor genomic differences could explain different phenotypes.

## Conclusion

Phenotypes variations were supported by genomic pairwise comparisons between Firmicutes and Bacteroidetes species. Overall, the analysis of Bacteroidetes genomes displayed two distinctive features, *i.e*. the antitoxin and CRISPR systems. On the other hand, the analysis of Firmicutes genomes allowed for the identification of multiple genes that could be involved in UV filters resistant mechanisms, coding for multidrug transporters, ROS responsive elements, periplasmic stress response regulons, or capsule components. Interestingly, bacteria with the bigger genomes held the resistant phenotypes, corroborating the fact that organisms with larger genomes are more adaptable to environmental and anthropogenic perturbations. While the presence of genes has been studied to decipher potential resistance mechanisms, the absence of a gene or operon could also be responsible for resistance by depleting the molecular target of UV filters. Further functional experiments such as transcriptomic and proteomic analyses would be needed to confirm the xenobiotic susceptibility and resistance biomarkers.

## Supplemental Information

10.7717/peerj.12278/supp-1 Supplemental Information 1 Function comparison_*Algoriphagus ornithinivorans VS Algoriphagus mannitolivorans*.

10.7717/peerj.12278/supp-2 Supplemental Information 2 Function comparison between *Bacillus megaterium VS Halobacillus dabanensis*.

10.7717/peerj.12278/supp-3 Supplemental Information 3 Function comparison between *Paenibacillus glucanolyticus VS Halobacillus dabanensis*.

## Additional Information and Declarations

### Data Availability

The following information was supplied regarding data availability: The genomic pairwise comparison obtained from the RAST platform (rast.nmpdr.org) are available in the Supplemental Files.
